# Targeting the PI3K/AKT pathway in prostate cancer: the role of PTEN deficiency and biomarker-guided therapy

**DOI:** 10.1080/15384047.2026.2694128

**Published:** 2026-06-26

**Authors:** Allison Staton, Melissa Abel, William D. Figg

**Affiliations:** a School of Pharmacy, Shenandoah University, Winchester, VA, USA; b NIH, Genitourinary Malignancies Branch, Center for Cancer Research, National Cancer Institute, Bethesda, MD, USA

**Keywords:** Prostate cancer, AKT inhibitor, PI3K/AKT, PTEN, biomarker

## Abstract

Loss of the *PTEN* tumor suppressor gene is a common molecular feature in advanced prostate cancer and is associated with activation of the PI3K/AKT signaling pathway, which controls cell growth and survival. Despite major advances in the treatment of metastatic castration-sensitive prostate cancer (mCSPC), patients with PTEN-deficient tumors represent an aggressive subgroup with poorer clinical outcomes and limited targeted therapeutic options. Capivasertib is a selective oral AKT inhibitor designed to suppress downstream signaling from PI3K pathway activation. We highlight the recent results from the Phase III CAPItello-281 trial (NCT04493853) demonstrating that the addition of capivasertib to abiraterone acetate and androgen deprivation therapy (ADT) significantly improved radiographic progression-free survival in patients with *PTEN*-deficient mCSPC, leading to the FDA-approval of this regimen. Notable adverse events in the cabavisertib arm included hyperglycemia, diarrhea, and rash. CAPItello-281 addresses a significant unmet need for *PTEN*-deficient mCSPC, suggesting AKT inhibition as a potential new targeted treatment strategy for a sub-population with poor prognosis.

Prostate cancer (PCa) is one of the most diagnosed cancers in men, accounting for approximately 30% of male diagnosed cancers in 2025.^1^ Metastatic castration-sensitive prostate cancer (mCSPC) accounts for approximately 15% of newly diagnosed cases in the Western hemisphere.^2^ Androgen deprivation therapy (ADT) remains the backbone of treatment for mCSPC. Combination regimens incorporating androgen receptor inhibitors (ARIs) or docetaxel have demonstrated significant improvements in overall survival compared to ADT alone.^3^ In addition, recent studies have shown benefit from combining ADT, docetaxel, and ARIs in select patients with high disease volume/and or risk.^4^ ^,^ ^5^ Notably, current first-line treatment strategies for mCSPC do not incorporate germline or somatic mutation testing or biomarker-guided therapy, raising the question of whether such an approach could further optimize outcomes.

The phosphoinositide 3-kinase (PI3K)/protein kinase B (AKT) pathway is a well-established therapeutic target in oncology and plays a major role in PCa progression. This signaling cascade regulates key cellular processes, including growth, proliferation, and resistance to apoptosis.^6^ Growth factors bind to cell-surface tyrosine kinase receptors, leading to PI3K activation and subsequent phosphorylation of AKT, which drives downstream signaling for growth and proliferation.^6^ The PI3K/AKT pathway is upregulated in approximately 50% of PCa, most commonly due to loss of phosphatase and tensin homolog (PTEN) function or PIK3/AKT genomic alterations.^7^ ^,^ ^8^

PTEN is a tumor suppressor protein that negatively regulates the PI3K/AKT pathway by dephosphorylating PI3K signaling intermediates, thereby inhibiting pathway activation. Approximately 50% of patients with metastatic prostate cancer (mPC) have PTEN mutations or copy number loss.^9^ Reduced PTEN expression has been consistently associated with more aggressive disease and poorer clinical outcomes.^10^ These observations have prompted an investigation into targeted therapies, such as AKT inhibitors. Preclinical PCa models showed enhanced tumor growth inhibition when both androgen receptor (AR) signaling and the PI3K/AKT pathway were targeted simultaneously, suggesting that patients with PTEN-deficient tumors may benefit from this dual inhibition strategy.^11^

Capivasertib is an oral, selective AKT inhibitor designed to target tumors with PI3K/AKT pathway activation. It is FDA-approved for HER2-negative, HR-positive breast cancer and is under investigation in PCa. Capivasertib is not the first agent in this class to be evaluated in PTEN-altered mPC. Ipatasertib, another AKT inhibitor, was investigated in metastatic castration-resistant prostate cancer (mCRPC) in the phase III IPATential150 trial (NCT03072238).^7^ This study, which showed a small but statistically significant benefit in radiographic progression-free survival (rPFS), formed the clinical basis for the hypothesis that combining AKT inhibition with AR pathway blockade could improve outcomes in patients with PTEN-deficient tumors. Building on these preclinical and clinical findings, the CAPItello-281 trial (NCT04493853), published in *ESMO Annals of Oncology*, was designed to evaluate whether capivasertib could provide a clinically meaningful benefit in patients with PTEN deficiency mCSPC.^12^

CAPItello-281 was a multicenter, global, double-blind, placebo-controlled phase 3 trial designed to evaluate the efficacy and safety of capivasertib plus standard of care versus placebo plus standard of care in patients with PTEN-deficient mCSPC.^12^ Standard of care consisted of abiraterone 1000 mg daily, prednisone, and ADT. A total of 1002 participants were randomized 1:1 to receive either capivasertib (400 mg twice daily for 4 d followed by 3 d off) plus standard of care, or placebo plus standard of care.^12^ Treatment was continued until progression of disease, toxicity, death, or discontinuation criteria defined by the protocol.

Eligible participants were males ≥ 18 y of age with de novo mCSPC, an Eastern Cooperative Oncology Group (ECOG) performance status of 0–1, and histologically confirmed adenocarcinoma with PTEN deficiency. Prior treatment with ADT, with or without abiraterone (plus prednisone or prednisolone), was permitted for up to 93 d before randomization, but patients who had received other prior therapies were excluded.^12^ PTEN status was assessed at screening by immunohistochemistry (IHC) and evaluated by a pathologist. PTEN deficiency was defined as ≥90% of malignant cells lacking cytoplasmic PTEN staining.^12^

The trial demonstrated a reduction in rPFS with capivasertib plus abiraterone compared with placebo plus abiraterone in the intention-to-treat population (median 33.2 vs 25.7 months; HR 0.81, 95% CI 0.66–0.98; *p* = 0.034).^12^ In addition, subgroup analyses showed the rPFS benefit was consistent, independent of baseline disease risk and metastatic disease volume. These results led to the FDA-approval of capavisertib with abiraterone and ADT for patients with PTEN-deficient mCSPC in June 2026. A post hoc subgroup analysis examining increasing PTEN deficiency (≥95%, ≥99%, and 100%) revealed that greater PTEN loss was associated with a greater reduction in disease progression for capivasertib plus abiraterone compared with placebo plus abiraterone. The hazard ratios at ≥95%, ≥99%, and 100% PTEN deficiency cutoffs were 0.75 (95% CI 0.60–0.94), 0.71 (95% CI 0.52–0.97), and 0.68 (95% CI 0.48–0.96), respectively, showing increased efficacy with higher levels of PTEN loss.^12^

Overall survival (OS), a secondary endpoint, was not statistically significant at the time of the primary analysis (HR 0.90, 95% CI 0.71–1.15; *p* = 0.401).^12^ Importantly, this analysis was performed at only 26% maturity, and further follow-up is planned to determine longevity benefits of treatment. Despite the lack of OS benefit at primary analysis, the trial demonstrated improvements in other secondary endpoints, including time to first subsequent therapy, symptomatic skeletal event–free survival, time to prostate-specific antigen progression, and time to castration resistance. Formal statistical testing was stopped after OS failed to reach significance. However, post hoc OS analyses at different PTEN deficiency cutoffs (≥95%, ≥99%, 100%) showed hazard ratios of 0.80 (95% CI 0.62–1.04), 0.77 (95% CI 0.53–1.12), and 0.77 (95% CI 0.51–1.14), respectively.^12^ This resembles the trend observed in rPFS, with increased benefit among patients with greater PTEN loss. Capivasertib was associated with higher rates of grade ≥ 3 adverse events (AE) (67% vs 40% with placebo), most commonly rash, hyperglycemia, and diarrhea. Deaths related to AEs were slightly higher in the capivasertib group (7.2% vs 5.2%), with causes spread out over many AEs, but none predominating.^12^ Overall, safety was consistent with prior studies, and no new signals were observed.

Similar to previous studies, the addition of AKT inhibitor to abiraterone and ADT demonstrates positive effects on PFS in mPC with PTEN loss, particularly in patients with the highest degree of PTEN deficiency.^7^ In IPATential150, the addition of ipatasertib to abiraterone improved PFS in the prespecified subgroup of patients with PTEN loss by IHC, a benefit that was not observed in the intention-to-treat population, which included patients irrespective of PTEN status. The trial provided several important insights that remain highly relevant in terms of biomarker testing. First, exploratory analyses suggested that patients with mutations in the PI3K/AKT/PTEN pathway, identified through next-generation sequencing (NGS), experienced greater PFS and OS benefit than patients classified solely by PTEN loss on IHC. Second, ipatasertib demonstrated a greater PFS benefit in patients with genomic PTEN loss (HR 0.65, 95% CI 0.45–0.95) or PIK3CA/AKT1/PTEN alterations identified by NGS (HR 0.63, 95% CI 0.44–0.88) compared with patients classified as having PTEN loss by IHC (HR 0.77, 95% CI 0.61–0.98).^7^ This suggests that NGS-based assessment better identifies patients who derive maximal benefit from AKT inhibition. For PCa, this implies that depending exclusively on IHC might underestimate the efficacy of targeted therapy in patients with PI3K/AKT/PTEN genomic alterations.

The importance of biomarker selection is further highlighted by the trial that led to FDA-approval of capivasertib in HER2-negative HR-positive breast cancer, where eligibility was determined using NGS-based detection of activating PIK3CA or AKT1 alterations and inactivating PTEN mutations, rather than IHC alone.^13^ Overall, this data supports a more extensive, genomics-driven approach to identifying PI3K/AKT pathway activation and suggest that more studies in mPC should move beyond IHC-defined PTEN loss toward more comprehensive molecular profiling.

Another relevant trial exploring AKT inhibition was CAPItello-280 (NCT05348577), which evaluated capivasertib in combination with docetaxel and ADT in an mCRPC population not selected for PTEN status.^14^ Prespecified secondary endpoints included analyses of PFS and OS in patients with PTEN-deficient and PTEN-proficient tumors. However, the trial was discontinued as the interim analysis predicted it would not meet primary endpoints, PFS and OS in overall mCRPC population. Although analyses of PFS and OS in patients with PTEN-deficient and proficient tumors were prespecified secondary endpoints, the termination of the study precluded reporting of these subgroup outcomes so far.^15^

The absence of a PFS and survival benefit in the overall CAPitello-280 study population does not exclude the possibility of therapeutic activity in defined subgroups. Prior studies, including CAPItello-281 and IPATential150, demonstrated that clinical benefit from AKT inhibition is largely selective for patients with PTEN-deficient disease, with exploratory analyses of IPATential150 showing greater PFS and OS benefit when PTEN deficiency or PI3K/AKT activation is defined using NGS rather than IHC alone. These observations raise the possibility that the negative outcome of CAPItello-280 reflects inclusion of patients without PI3K/AKT pathway activation, rather than a lack of biological activity of AKT inhibition in mCRPC. Overall, the data suggest that capivasertib may be most effective as a targeted therapy in selected populations, underscoring the need for biomarker-driven trial designs and standardized molecular definitions (NGS vs IHC) in future studies, particularly for early treatment of mPC.

Nonetheless, with long-term survival data still pending, the clinical application of capivasertib remains limited, especially in mCSPC. However, CAPItello-281 shows the potential of biomarker- and genomics-driven strategies to identify a subset of patients with poor-prognosis disease who may benefit from targeted therapy. This evidence raises the important question of whether PTEN deficiency and PI3K/AKT mutations should be incorporated into clinical guidelines for mPC to guide precision therapy and optimize patient outcomes.

## Data Availability

This article does not require data.
